# Upregulation of CD94 on CD8^+^T Cells in Anterior Chamber-Associated Immune Deviation

**DOI:** 10.1186/1471-2172-9-53

**Published:** 2008-09-25

**Authors:** Hao He, Peizeng Yang, Liqiong Jiang, Junfeng Zhang, Changlin Zhao, Lina Chen, Xiaomin Lin, Hongyan Zhou, Aize Kijlstra

**Affiliations:** 1 State Key Laboratory of Ophthalmology, Zhongshan Ophthalmic Center, Guangzhou, PR China; 2 The First Affiliated hospital, Chongqing Medical University, Chongqing, PR China; 3 Eye Research Institute Maastricht, Department of Ophthalmology, University Hospital Maastricht, Maastricht, The Netherlands; 4 Animal Sciences Group, Wageningen University, Lelystad, The Netherlands

## Abstract

**Background:**

CD8^+ ^regulatory T cells (Treg) have been considered to be involved in a model of ocular-induced tolerance, known as anterior chamber-associated immune deviation (ACAID). The phenotype and characteristics of CD8^+^Treg in ACAID remain only poorly understood. Recent studies have reported that the CD94-Qa-1 system is implicated in the induction of ACAID CD8^+^Treg, but the functions and characteristics of CD8^+^CD94^+^T cells remain unclear.

**Results:**

Both mRNA and protein of CD94 and NKG2A were markedly up-regulated on splenic CD8^+^T cells of ACAID mice compared with controls. Flow cytometric analysis showed that very few CD8^+^CD94^+^T cells express granzyme B, perforin and Foxp3. CD8^+^CD94^+^T cells, but not CD8^+^CD94^-^T cells, magnetically isolated from the spleens of ACAID mice, produced large amounts of TGF-beta1 and exhibited suppressive activity in vitro. Neutralization of TGF-beta1 caused reversal of suppression mediated by CD8^+^CD94^+^T cells.

**Conclusion:**

CD8^+^CD94^+^T cells from ACAID mice exhibited suppressive activity in association with enhanced expression of TGF-beta1, suggesting that CD8^+^Treg are mainly distributed in CD94^+^T cell subpopulations.

## Background

Injection of soluble antigens (Ag) into the anterior chamber (AC) of the eye induces systemic tolerance known as anterior chamber-associated immune deviation (ACAID). It is characterized by an antigen-specific suppression of the delayed-type hypersensitivity (DTH) response and a shift in antibody production away from complement-fixing isotypes[1,2]. At least four organs or tissues including an intact eye, spleen, thymus and sympathetic nervous system are involved in the induction of ACAID, which eventually generates two distinct regulatory T(Tregs) cell populations in the spleen[3]. One population of Tregs comprises CD4^+^T cells which function as afferent suppressor cells and suppress the induction of a DTH response. The other Treg cell population consists of CD8^+^T cells which mediate an efferent suppression and inhibit the expression of a DTH response. However, little is known about the phenotype of CD8^+^Tregs in ACAID.

The CD94 molecule belongs to the C-type lectin superfamily and forms a functional heterodimer with different subtypes of NKG2 [4-6]. The CD94-NKG2 receptor was originally discovered in NK cells and subsequently found on different T cell subsets, especially CD8^+^T cells[7,8]. The ligand for these receptors was the nonclassical class I molecule, Qa-1 in mice, and its homologue HLA-E in humans [9-11]. By binding to different NKG2 subsets, CD94 could regulate the cytotoxicity and survival of NK cells and CD8^+^T cells upon reaction with their ligand [12-15]. Recently, a few studies reported that CD94 was associated with the regulatory function of NK cells and CD8^+^T cells. Della Chiesa and coworkers[16] found that a NK cell subset expressing CD94-NKG2A in lymph nodes was capable of killing immature DC and mature DC to prevent the overactivation of DCs. Using DNA microarray analysis, Keino et al[17] compared patterns of gene expression in regulatory OT-1 CD8^+^T cells generated in vitro with those from nonregulatory OT-1 CD8^+^T cells. They found that CD94 mRNA was up-regulated in OT-1 CD8^+^Tregs, suggesting that CD94 may play a role in immune regulation by Tregs. Chattopadhyay et al[18] extended this study and reported that mice with deficiency of CD94-NKG2A were unable to develop ACAID, and CD8^+^T cells from these mice did not transfer the local suppression of a DTH response. Their study indicated that the CD94 molecule is necessary for the immunosuppression mediated by CD8^+^Treg in ACAID. However, the suppressive mechanism and characteristics of CD8^+^CD94^+^T in ACAID remain unclear. The present study showed that CD94 was up-regulated on CD8^+^T cells from the spleens of ACAID mice. Very few CD8^+^CD94^+^T cells express granzyme B, perforin and Foxp3. CD8^+^CD94^+^T cells, but not CD8^+^CD94^-^T cells from ACAID mice exhibited suppressive activity in association with enhanced expression of TGF-beta1. These results suggest that CD8^+^Treg in ACAID are mainly distributed in CD94^+^T cell subpopulations.

## Results

### Upregulation of the CD94-NKG2A receptor on splenic CD8^+^T cells of ACAID mice

**Figure 1:**
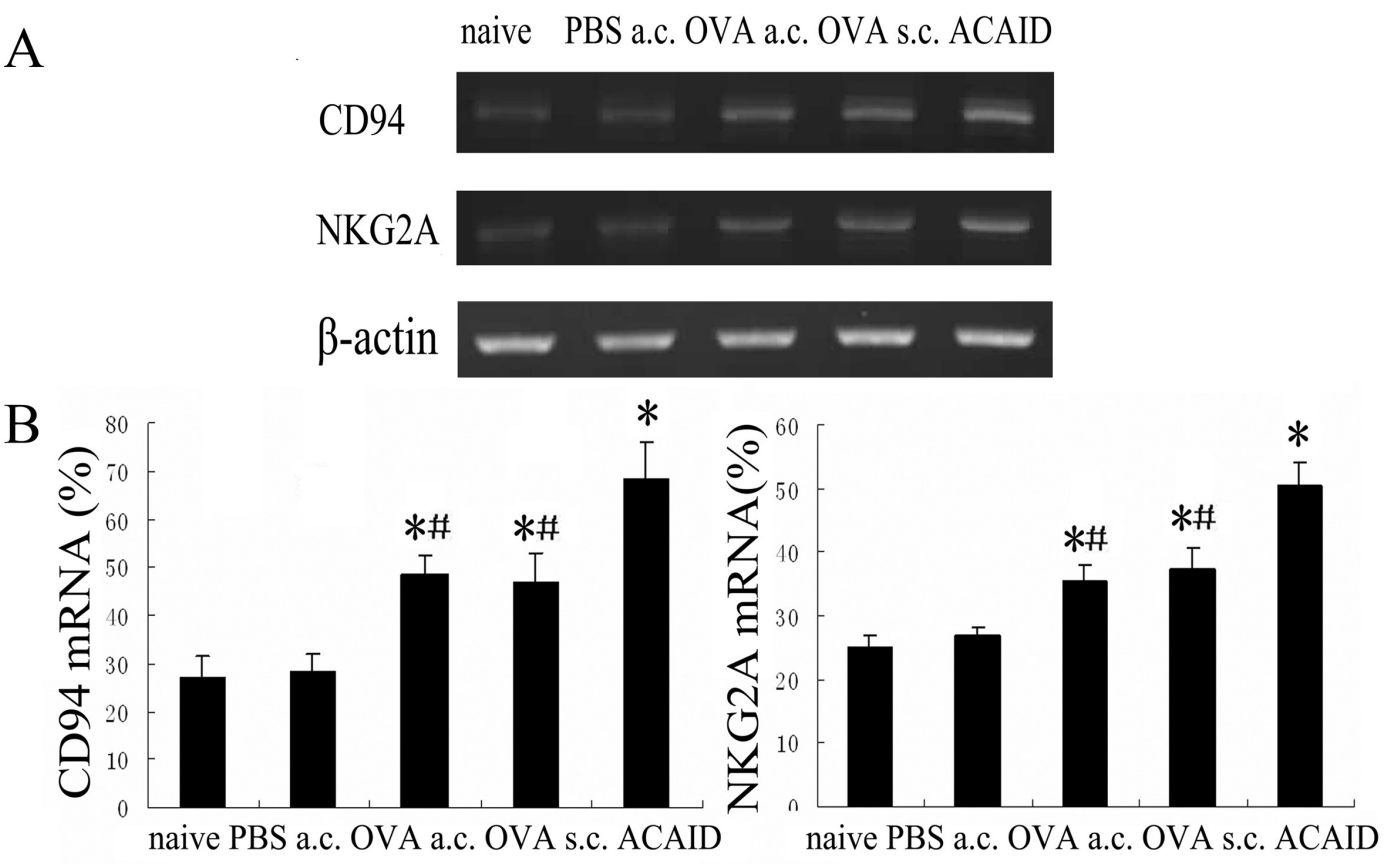
**Expression of CD94-NKG2A mRNA on CD8^+^T cells**. Mice underwent various treatments and were divided into naïve group, PBS-AC-injected group, OVA-AC-injected group, immunized group and ACAID group. A: CD8^+^T cells magnetically sorted from splenic MNC of each group were lyzed to obtain total cellular RNA using TRIzol, and RT-PCR was performed to analyze CD94 and NKG2A mRNA expression. B: histograms showed the mRNA expression (mean percentage ± SEM) relative to β-action expression (set at 100%). The results shown are representative of three independent experiments. *p < 0.05 compared with naïve mice. ^#^p < 0.05 compared with ACAID mice.

**Figure 2:**
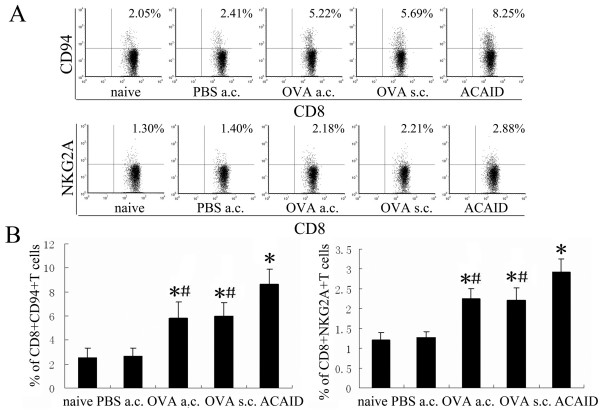
**Expression of CD94-NKG2A protein on CD8^+^T cells**. Mice underwent various treatments and were divided into naïve group, PBS-AC-injected group, OVA-AC-injected group, immunized group and ACAID group. A: Splenic MNC were harvested from each group and stained with anti-CD3, anti-CD8, anti-CD94 and anti-NKG2A mAb, then the CD3^+^CD8^+^T population was gated and analyzed for the expression of CD94 and NKG2A protein. B: FCM histogram showed the frequencies of CD8^+^CD94^+^T and CD8^+^NKG2A^+^T cells among CD8^+^T cells. Results represent the mean ± SEM of three separate experiments. *p < 0.05 compared with naïve mice. ^#^p < 0.05 compared with ACAID mice.

RT-PCR and flow cytometry were used to investigate the expression of CD94 and NKG2A on CD8^+^T cells at the mRNA and protein level during ACAID. As shown in Figure 1, mRNA of CD94 and NKG2A was detectable on CD8^+^T cells in all groups, but it was significantly higher in ACAID mice as compared to immunized mice, OVA-AC-injected mice, PBS-AC-injected mice and naïve mice. The expression of these two molecules in immunized mice and OVA-AC-injected mice was also significantly higher than that observed in PBS-AC-injected mice and naïve mice. As shown in Figure 2, the frequencies of CD94 and NKG2A expression on CD8^+^T cells from spleens were significantly higher in ACAID mice(8.68%, 2.92%) compared with immunized mice(6.0%, 2.2%), OVA-AC-injected mice(5.84%, 2.25%), PBS-AC-injected mice(2.65%, 1.27%) and naïve mice(2.56%, 1.21%). The OVA-AC-injected mice and immunized mice also showed significantly increased frequencies of splenic CD8^+^CD94^+^T and CD8^+^NKG2A^+^T cells compared with PBS-AC-injected mice and naive mice.

### Expression of perforin, granzyme B and Foxp3 in CD8^+^CD94^+^T cells was minimal

**Figure 3:**
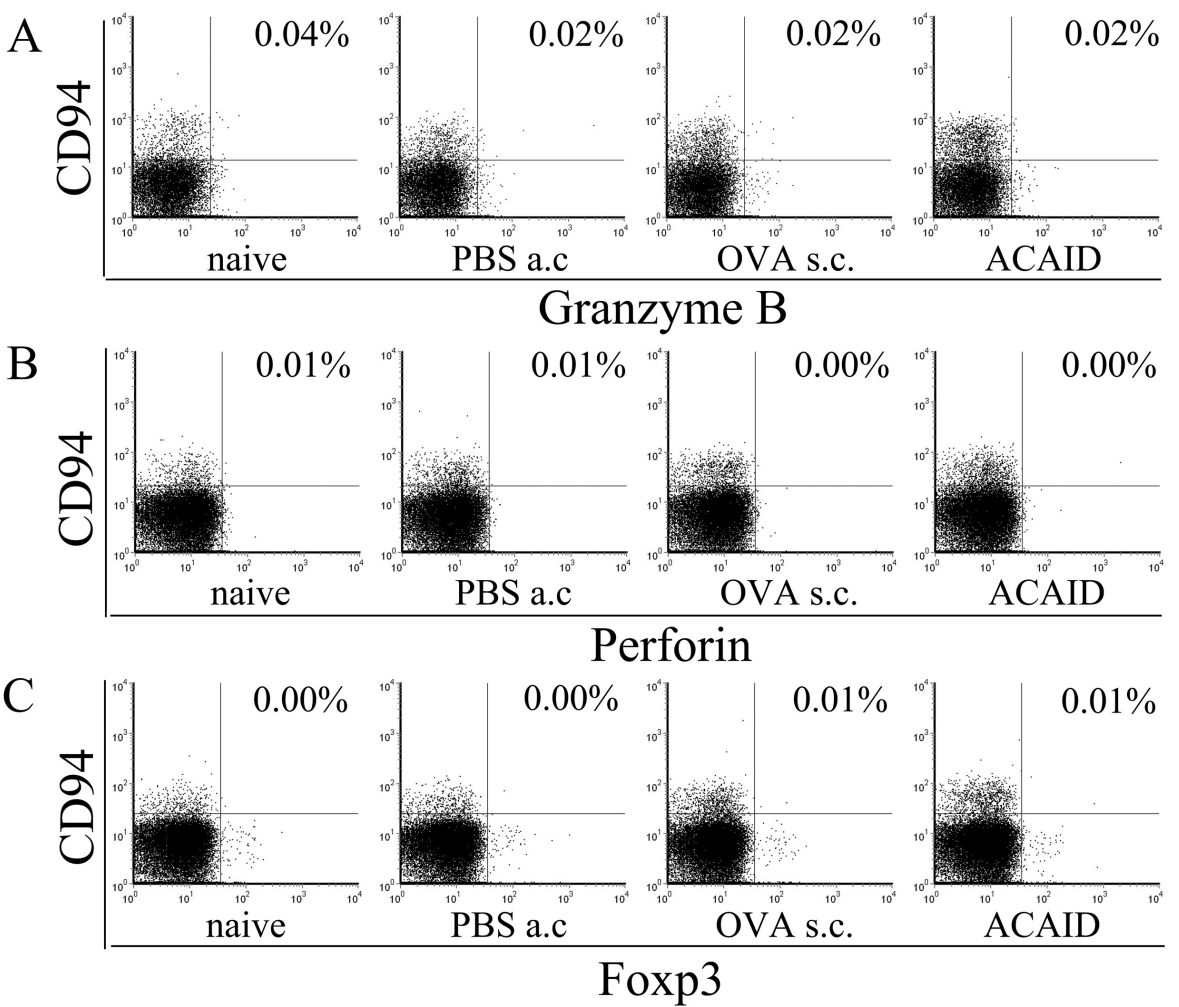
**Expression of granzyme B, perforin and Foxp3 in CD8^+^CD94^+^T cells**. Splenic MNC from ACAID mice, immunized mice, PBS-AC-injected mice and naïve mice were prepared and labeled with anti-CD3, anti-CD8, anti-CD94, anti-perforin, anti-granzyme B and anti-Foxp3 mAb. A: CD3^+^CD8^+^T population was gated and analyzed for the frequency of CD94^+^granzyme B^+^T cells. B: CD3^+^CD8^+^T population was gated and analyzed for the frequency of CD94^+^perforin^+^T cells. C: CD3^+^CD8^+^T population was gated and analyzed for the frequency of CD94^+^Foxp3^+^T cells. The results shown are representative of three independent experiments.

To characterize CD8^+^CD94^+^T cells, we examined the intracellular expression of granzyme B, perforin and Foxp3. As shown in Figure 3, the expression of intracellular granzyme B, perforin and Foxp3 in fresh CD8^+^CD94^+^T cells was minimal(~0.04%, ~0.01% and ~0.01%) in ACAID mice, immunized mice, PBS-AC-injected mice and naïve mice. There was no significant difference among different groups.

### CD8^+^CD94^+^T cells from ACAID mice exhibited suppressive activity in vitro

**Figure 4:**
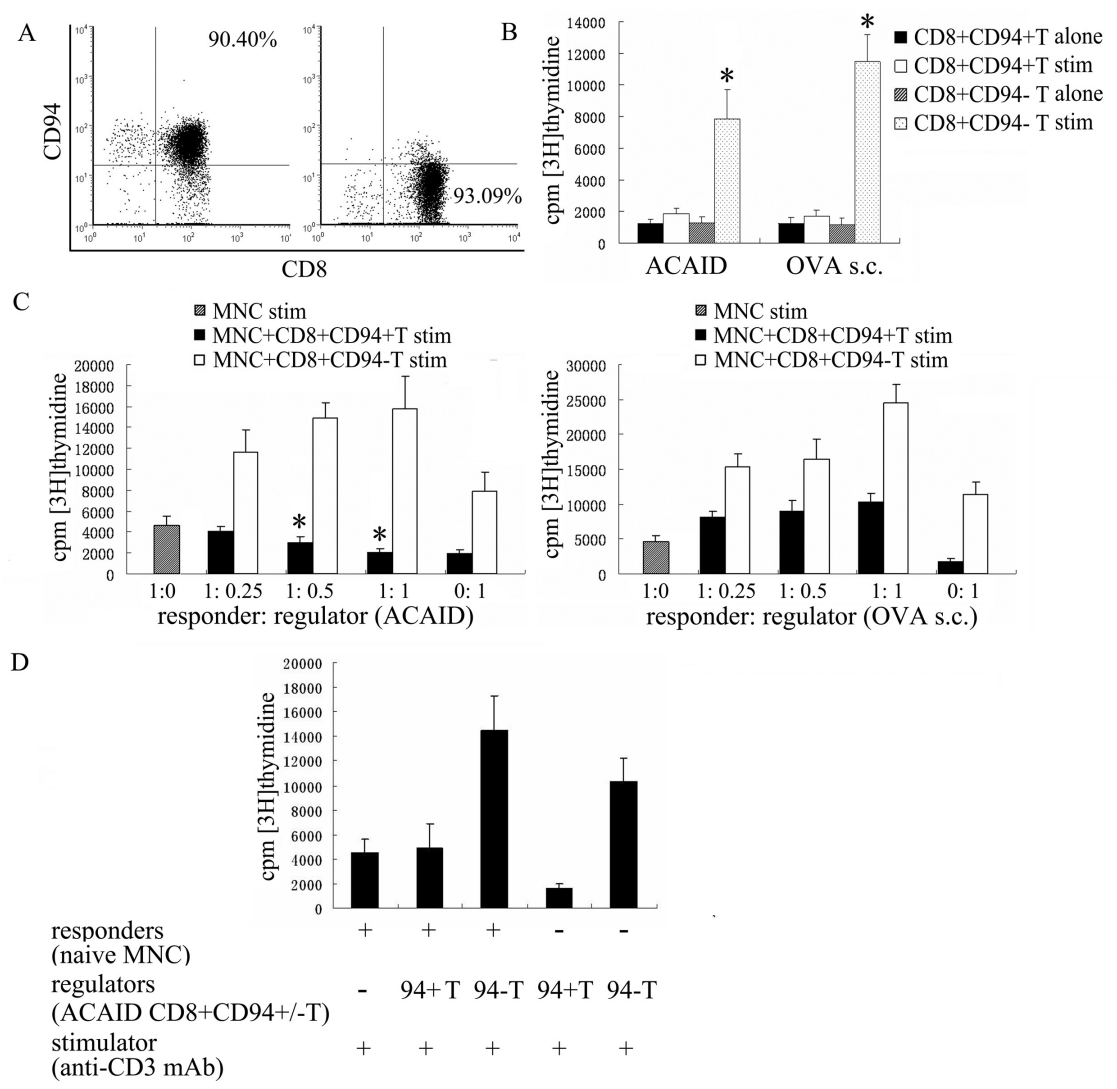
**Suppressive activity exhibited by CD8^+^CD94^+^T cells from ACAID mice in vitro**. A: the purity of isolated CD8^+^CD94^+^T (left) and CD8^+^CD94^-^T (right) cells by magnetic affinity cell sorting; B: the response of CD8^+^CD94^+^T and CD8^+^CD94^-^T cells to anti-CD3 mAb stimulation (stim). *p < 0.05 compared with unstimulated group. C: suppressive effect of CD8^+^CD94^+^T and CD8^+^CD94^-^T cells on the proliferation of splenic MNC. Splenic MNC (5 × 10^4 ^cells/well, as responder cells) from immunized mice were cocultured with purified CD8^+^CD94^+^T or CD8^+^CD94^-^T cells (as regulatory cells) from ACAID mice and immunized mice at different responder/suppressor ratios (1:0, 1:0.25, 1:0.5 and 1:1) in the presence of OVA(100 μg/ml) and anti-CD3 mAb(1 μg/ml) for 72 hours and pulsed with [3H]thymidine for the last 16 hours of culture. Data are represented as mean ± SEM of triplicate samples. *p < 0.05 compared with stimulated MNC group. D: suppressive effect of CD8^+^CD94^+^T and CD8^+^CD94^-^T cells from ACAID mice on the proliferation of splenic MNC from naive mice (at 1:1 ratio) upon anti-CD3 mAb(1 μg/ml) stimulation.

Splenic CD8^+^CD94^+^T and CD8^+^CD94^-^T cells were magnetically sorted from ACAID mice and immunized mice and were shown to be over 90% pure (Fig. 4A). Purified CD8^+^CD94^+^T cells from two groups displayed hyporesponsiveness to stimulation with anti-CD3 mAb, whereas CD8^+^CD94^-^T cells proliferated strongly (Fig. 4B). We next investigated whether these two subsets had suppressive effect on the proliferation of responder cells when exposed to OVA and anti-CD3 mAb in vitro. As shown in Figure 4C, CD8^+^CD94^+^T cells, but not CD8^+^CD94^-^T cells, from ACAID mice showed a significantly suppressive effect on the proliferation of splenic mononuclear cells (MNC) from mice with conventional immunization in a dose-dependent manner. However, both CD8^+^CD94^+^T cells and CD8^+^CD94^-^T cells from immunized group did not exhibit this suppression. Additionally, OVA-AC-induced CD8^+^CD94^+^T cells did not influence the proliferation of splenic MNC from naive mice when exposed to the anti-CD3 mAb stimulation (Fig. 4D).

### Suppressive activity of CD8^+^CD94^+^T cells in ACAID was associated with TGF-beta1 production

**Figure 5:**
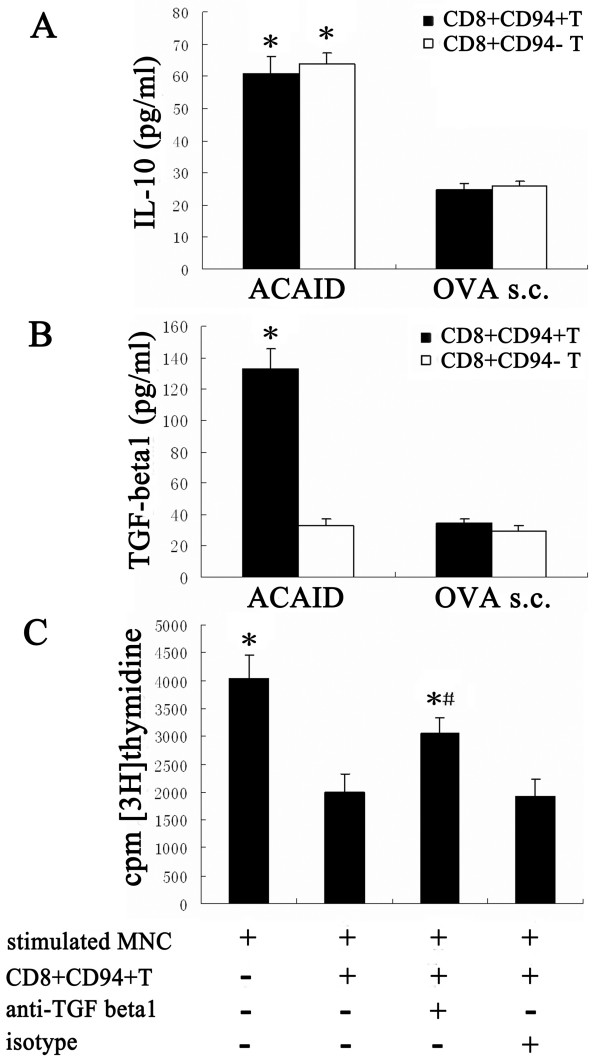
**IL-10 and TGF-beta1 production and the role of TGF-beta1 in immunosuppression**. CD8^+^CD94^+^T or CD8^+^CD94^-^T cells(5 × 10^4^) from ACAID mice and immunized mice were cocultured with PEC(1 × 10^5^) and OVA (100 μg/ml) for 72 hours. ELISA was used to detect the concentration of IL-10 (A) and TGF-beta1 (B) in the supernatants of the cultures. *p < 0.05 compared with immunized mice. For blocking experiments (C), anti-TGF-beta1 mAb(50 μg/ml) or matched isotype was added to the culture at the initiation of the cell proliferation assay, then [3H]thymidine incorporation was measured to value the suppressive activity of CD8^+^CD94^+^T cells from ACAID mice. Data are represented as mean ± SEM of triplicate samples. *p < 0.05 compared with stimulated MNC+CD8^+^CD94^+^T cell group (the second bar). ^#^p < 0.05 compared with stimulated MNC group (the first bar).

An ELISA was used to measure IL-10 and TGF-beta1 in the supernatants of the cultures containing APC, OVA, CD8^+^CD94^+^T or CD8^+^CD94^-^T cells. The results showed that both CD8^+^CD94^+^T and CD8^+^CD94^-^T cells in ACAID mice could equally produced a large amount of IL-10, which was significantly higher than that in mice following conventional immunization (Fig. 5A). Interestingly, CD8^+^CD94^+^T cells from ACAID mice secreted larger amounts of TGF-beta1 as compared with CD8^+^CD94^-^T cells from these mice. TGF-beta1 production from the conventionally immunized control mice was also much lower than that observed by CD8^+^CD94^+^T cells from ACAID mice (Fig. 5B). A blocking experiment with anti-TGF-beta1 mAb was performed to examine its influence on the cell proliferation. As shown in Figure 5C, neutralization of TGF-beta1 was able to decrease the CD8^+^CD94^+^T cell suppressive activity by 27.4%. There was still significant difference between CD8^+^CD94^+^T-untreated group and TGF-beta1-neutralization group.

## Discussion

The present study showed that expression of CD94 was up-regulated on CD8^+^T cells during ACAID. CD8^+^CD94^+^T cells were characterized by minimal expression of perforin, granzyme B and Foxp3. Furthermore the study showed that the CD8^+^CD94^+^T subpopulation from ACAID mice could significantly inhibit splenic MNC proliferation in vitro and that these cells produced large amounts of TGF-beta1. These results suggest that the CD8^+^CD94^+^T subpopulation play a role in the immune regulation.

Although CD94 could pair with different NKG2 molecules, most CD94/NKG2 receptors on both NK and CD8^+^T cells have been shown to be CD94-NKG2A heterodimer in mice [8,11]. We first examined the expression of CD94 and NKG2A on splenic CD8^+^T cells. The results showed that a significantly higher expression of CD94-NKG2A heterodimer on CD8^+^T cells was induced following OVA inoculation into the anterior chamber, and that AC-injection of antigen and immunization have a synergistic effect on the increase of CD8^+^CD94^+^T cells. Given the important role of cytokines in CD94-NKG2A expression [19-23], this upregulated receptor may be due to the promotion of certain cytokines from the splenic immunomodulatory microenvironment during the induction of ACAID. Interestingly, we also found an increased expression of CD94-NKG2A in mice following conventional immunization. This result is in agreement with the results presented by Jabri et al[24]. They found that CD94-NKG2A could be induced on CD8^+^T cells following TCR-mediated activation in vivo.

It is well known that CTL, which are mainly found in the CD8^+^T cell subpopulation, are impaired during ACAID[25]. Analysis of the expression of perforin and granzyme B, two important molecules for cytotoxicity, on CD8^+^CD94^+^T only revealed a moderate expression. These data are consistent with an earlier study we reported, where we could not detect secretion of granzyme B by CD8^+^T cells in ACAID mice, whereas production in control immunized mice was readily observed [26]. This phenomenon is also confirmed by Cone et al that perforin was not required for suppression by ACAID CD8^+^Treg[27]. The very low production of granzyme B and perforin by CD8^+^CD94^+^T cells in each group in this study suggests that these two molecules are not involved in the development of ACAID.

Although CD8^+^T cells from ACAID mice exert suppression[28,29], it is unknown that which subpopulation of CD8^+^T cells exhibit suppressive activity. Chattopadhyay[18] et al demonstrated that DBA/2J mice, characterized by a deficiency in CD94/NKG2A, showed a defective immune regulation by CD8^+^Treg response in vivo following intracameral antigen inoculation. Their study supported that CD94 is necessary for the generation and function of CD8^+^Treg, but no results about the suppressive activity of CD8^+^CD94^+^T cells are showed. As both intracameral inoculation and conventional immunization could upregulate CD94-NKG2A expression, we examined the inhibitory effect of CD8^+^CD94^+^T cells from ACAID mice and immunized mice on the proliferation of splenic MNC in vitro. The results showed that only CD8^+^CD94^+^T cells from ACAID mice profoundly inhibited the proliferation of responder cells, CD8^+^CD94^-^T cells from ACAID mice as well as immunized mice showed no suppressive activity. As OVA and anti-CD3 mAb was concurrently used to stimulate MNC from the immunized mice, both antigen-specific and non-specific suppression were measured in our system. In a pilot experiment we tried to detect the antigen-specific suppression by using OVA alone. However, a weak response of MNC to antigen made it difficult to compare the result between positive and suppression groups. In contrast, we found that OVA-AC-induced CD8^+^CD94^+^T cells did not influence the proliferation of splenic MNC from naive mice when exposed to polyclonal stimulation, this result seems to suggest a property of antigen-specific suppression of CD8^+^CD94^+^T cells in ACAID. It is consistent with the previous observations that CD8^+^T cells in ACAID are efferent antigen-specific Tregs. Another property of CD8^+^CD94^+^T cells was hypoproliferation when stimulated with polyclonal stimulation, this anergic state of CD8^+^CD94^+^T cells was similar to other Tregs such as CD4^+^CD25^+^Treg[30,31]. However, CD8^+^CD94^-^T cells did not show any suppressive activity and anergy, suggesting the non-Treg's property of these cells. These data suggest that the induced expression of CD94 molecule is associated with the inhibitory effect of CD8^+^T cells following OVA inoculation into the anterior chamber.

It is interesting that CD8^+^CD94^+^T cells from ACAID mice, but not from immunized mice exert suppression in vitro. What results in the different suppressive activity of CD8^+^CD94^+^T cells between tolerant mice and immunized mice? As cells exert their function mainly through releasing cytokines or contacting the target cells, it is possible that different cytokine secretion may cause different functional effects. IL-10 and TGF-beta have been shown to be the suppressive cytokines of CD8^+^Treg in some models [32-39]. Wang and collaborators found that an increased IL-10 and TGF-beta2 were observed in the cultures containing AC-splenic T cells, lymph node cells and antigen[28]. Kezuka et al[40] demonstrated that TGF-beta2 is not required for the suppression mediated by in vitro-activated OT-1 Treg, similar to ACAID CD8^+^Treg. We subsequently analyzed the production of TGF-beta1 and IL-10 to evaluate the association of these inhibitory cytokines with suppressive property of CD8^+^CD94^+^T cells. Our result showed that CD8^+^CD94^+^T cells from ACAID mice produced larger amounts of TGF-beta1 and IL-10 than those from conventionally immunized mice, suggesting that different cytokine secretion is associated with different inhibitory property. It is interesting to note that there was no difference between CD8^+^CD94^+^T cells and CD8^+^CD94^-^T cells concerning IL-10 production. The unique increased TGF-beta1 production by the CD8^+^CD94^+^T cells with suppressive activity in ACAID mice raises a question as to whether these cells exert their role via TGF-beta1. Our further experiment using anti-TGF-beta1 antibody tested this possibility and found that it could partially block the inhibitory activity of CD8^+^CD94^+^T cells. The anti-TGF-beta1 antibody has been titrated to test whether it could completely neutralize its inhibition. Even higher concentrations still showed a partial inhibition (data not shown). It has been demonstrated that different subpopulations of CD8^+^Treg exert their immunosuppression possibly by different mechanisms. There may be other factors involved in this inhibition, for instance cell-cell contact, other isoforms of TGF-beta or other cytokines. More studies are needed to clarify these issues.

Given the different suppressive activity between CD8^+^CD94^+^T and CD8^+^CD94^-^T cells in ACAID mice, we thinks that CD8^+^Treg are mainly distributed in CD94^+^T subpopulation in ACAID. However, it appears that CD94 is not a specific marker for CD8^+^Treg, as this molecule can be upregulated following T cell activation and CD8^+^CD94^+^T cells from immunized mice did not show suppression. This phenomenon resembles the role of CD25, CTLA-4 and GITR in identifying CD4^+^Treg. What is the specific marker for CD8^+^Treg? Recent studies reported that one population of CD8^+^Tregs was associated with Foxp3 expression [41-43], we further examined the expression of Foxp3 by CD8^+^CD94^+^T cells. Our data showed that very few CD8^+^CD94^+^T cells expressed Foxp3 and there was no significant difference among different experimental groups. Earlier studies from our group also showed a very low expression of Foxp3 by CD8^+^T cells, but the frequency of CD8^+^Foxp3^+ ^cells in splenocytes of ACAID mice was upregulated following polyclonal or specific antigen stimulation in vitro[29]. In the present study, splenocytes were not treated with any stimulation, which may explain why we did not detect any significant difference in Foxp3 expression among the different groups. Our results support the opinion provided by Ahmadzadeh et al[44] that Foxp3 can not be simply interpreted as an indicator of Treg activity. Our study also suggests that CD8^+^CD94^+ ^T cells may be only one population of CD8^+ ^Treg.

## Conclusion

The data presented in this paper showed that CD8^+^CD94^+^T cells from ACAID mice exhibited suppressive activity in association with enhanced expression of TGF-beta1, suggesting CD8^+^Treg may be mainly distributed in CD94^+^T subpopulation in ACAID. However, it appears that CD94 is not a specific marker for CD8^+^Treg, because this molecule is associated with CD8^+^T cell activation and no suppression was observed by CD8^+^CD94^+^T cells from immunized mice. There are also some limitations in our study. The profile of cytokines by CD8^+^CD94^+^T remains unclear. Due to the low number of CD8^+^CD94^+^T cells from ACAID mice, we were not able to perform adoptive transfer experiments. More studies using more sensitive techniques and more experiments are needed to clarify the role of CD8^+^CD94^+ ^T cells in the development of ACAID. As NKT cells may be positive for both CD8 and CD94 molecules [45-47], the isolated CD8^+^CD94^+ ^T cells in this study may contain NKT cells.

## Methods

### Mice

Female C57BL/6 (B6; H-2b) mice, 6 to 8 weeks of age, were purchased from the animal facility at the Sun Yat-Sen University, P.R. China. All mice were treated according to the ARVO Statement for the Use of Animals in Ophthalmic and Vision Research.

### Induction of ACAID

ACAID was induced as described previously using microinjection of antigen into the AC of the eye[48]. Briefly, mice were anesthetized with inhalation anesthesia consisting of oxygen and 1.7% isoflurane followed by injection of 5 μl ovalbumin at 20 mg/ml (OVA, Sigma, St. Louis, MO) into the AC by a glass micropipette with a sterile infant feeding tube mounted onto a 0.1 ml Hamilton (Hamilton, Reno, NV) syringe. Seven days later, mice received a subcutaneous (s.c.) immunization with 250 μg OVA dissolved in PBS and emulsified in complete Freund's adjuvant (Life Technologies, Grand Island, NY). Mice only receiving subcutaneous injections of OVA in CFA were used as immunized controls (OVA s.c.). Mice receiving an AC-injection of 5 μl sterile PBS alone served as PBS-AC-injected controls (PBS a.c.). Mice receiving an AC-injection of 5 μl OVA without subsequent immunization served as OVA-AC-injected controls (OVA a.c.).

### Preparation of peritoneal exudate cells

Peritoneal exudate cells (PECs) were obtained from normal C57BL/6 mice that received 2 ml of thioglycolate (Sigma Chemical Co.) intraperitoneally three days earlier. Briefly, the recovered cells were washed and resuspended, placed in a 24-well culture plate (1 × 10^6^/well), and incubated in complete RPMI 1640 medium at 37°C in an atmosphere of 5% CO_2_. After overnight culture, plates were washed three times with culture medium to remove nonadherent cells. Adherent cells were retained in the wells and used as antigen-presenting cells (APC). More than 90% of these adherent cells were F4/80^+ ^as identified by subsequent FCM analysis.

### Reverse transcription-PCR

Total cellular RNA was isolated from 5 × 10^6 ^freshly purified splenic CD8^+^T cells of the experimental and control mice using Trizol (Invitrogen, San Diego, CA). Single-strand cDNA was synthesized with an RT-PCR kit (Qiagen, Hilden, Germany) according to the manufacturer's instructions. The following primers were used: CD94-FW, 5'-TTTCTTGATGGTTACTTTGGGAGTT-3'; CD94-RV, 5'-AAACGCTTTTGCTTGGACTGTA-3'; NKG2A-FW, 5'-CAGTCATCGAGCAGGAAATC-3'; NKG2A-RV, 5'-GCTGACCTCTGCCCTTCCGA-3'; β-actin-FW, 5'-GTCCCTCACCCTCCCAAAAG-3'; β-actin-RV, 5'-GCTGCCTCAACACCTCAACCC-3'. PCR was performed with a initially heating to 94°C for 5 min followed by 30 cycles at 94°C for 30 s, 60°C for 30 s, and 72°C for 60 s. Splenocytes from four mice were used in each group in one experiment. The experiment was repeated three times.

### Flow cytometry

For cell surface staining, splenocytes(1 × 10^6^) were stained with FITC or PE-Cy7-anti-CD3 mAb, APC or FITC anti-CD8 mAb, PE anti-CD94 mAb, PE-anti-NKG2A mAb for 30 minutes at 4°C in the dark. For intracellular cytokine staining, cells were first stained for surface markers followed by fixation and permeabilization, and were then stained with APC-anti-perforin mAb, FITC-anti-granzyme B mAb, APC-anti-Foxp3 mAb or matched isotypes according to the manufacturer's instructions. Phenotypic analysis was performed on a BD FACSAria(BD Biosciences, San Jose, CA) using the BD FACSDiVa software (BD Biosciences). All conjugated antibodies were purchased from eBioscience(San Diego, CA). Four mice were used for each group in one experiment. These experiments were repeated three times.

### Magnetic affinity cell sorting

Splenic mononuclear cells (MNC) were prepared by density gradient centrifugation. CD8^+^T cells were purified from splenic MNC of C57BL/6 mice by negative selection using a CD8^+^T-cell isolation kit (Miltenyi Biotec, Auburn, CA) according to the manufacture's instructions. Enriched CD8^+^T cells were stained with PE-conjugated anti-mouse CD94 mAb (eBioscience, San Diego, CA) for 30 minutes at 4°C in the dark followed by anti-PE microbeads (Miltenyi Biotec) for 15 minutes at 4°C. A positive selection was performed and both CD8^+^CD94^+^T and CD8^+^CD94^-^T cells were isolated as described by the manufacturer. The purity of CD8^+^CD94^+^T and CD8^+^CD94^-^T cells was > 90% as determined by subsequent FCM analysis.

### Proliferation and cytokine assays

Splenic MNC (5 × 10^4 ^cells/well, as responder cells) from immunized mice were cocultured with purified CD8^+^CD94^+^T or CD8^+^CD94^-^T cells (as regulatory cells) from ACAID mice and immunized mice at different responder/suppressor ratios (1:0, 1:0.25, 1:0.5, 1:1) in the presence or absence of OVA(100 μg/ml) and anti-CD3 mAb(1 μg/ml) (eBioscience, San Diego, CA) for 72 hours and pulsed with [3H]thymidine (Shanghai Institute of Applied Physics, Chinese Academy of Sciences, China) for the last 16 hours of culture. [3H]thymidine incorporation was determined by scintillation spectrometry. The suppressive effect of CD8^+^CD94^+^T and CD8^+^CD94^-^T cells from ACAID mice on the proliferation of splenic MNC from naive mice (at 1:1 ratio) upon anti-CD3 mAb(1 μg/ml) stimulation was examined. In addition, the proliferation of CD8^+^CD94^+^T and CD8^+^CD94^-^T cells (5 × 10^4 ^cells/well) from ACAID mice and immunized mice to the polyclonal stimulation was also examined. In order to clarify whether TGF-beta1 has an effect on suppression mediated by CD8^+^CD94^+^T cells from ACAID mice, additional proliferation assays were performed after blocking with anti-TGF beta1 mAb or matched isotype(R&D Systems, Inc, USA). Proliferation data were expressed as mean cpm ± SEM of triplicate reactions. To detect IL-10 and TGF-beta1 production, freshly isolated splenic CD8^+^CD94^+^T or CD8^+^CD94^-^T cells from various groups of mice were cultured in the 96-well plates containing PECs(1 × 10^5^/well) and OVA(100 μg/ml) for 72 hours. Supernatants were collected and assayed by ELISA (R&D system, Minneapolis, MN, USA) according to the manufacturer's instructions. Four mice were used for each group in one experiment. The experiment was repeated three times.

### Statistical analyses

All statistical analyses were performed by ANOVA using SPSS 11.0. A value of p < 0.05 was considered significantly different.

## Authors' contributions

HH carried out the whole studies and drafted the manuscript. PY was responsible for the overall design and execution of the experimental program. LJ and JZ were responsible for induction of ACAID. CZ and LC participated in the flow cytometric analysis and ELISA. XL and HZ performed in PT-PCR and cell proliferation assay. AK contributed to date interpretation and manuscript revision. All authors have read and approved the final manuscript.
